# The potential climatic range of spotted lanternfly may be broader than previously predicted

**DOI:** 10.3389/finsc.2023.1092189

**Published:** 2023-01-30

**Authors:** Melody A. Keena, George Hamilton, Devin Kreitman

**Affiliations:** ^1^Northern Research Station, United States Department of Agriculture (USDA) Forest Service, Hamden, CT, United States; ^2^Department of Entomology, Rutgers, The State University of New Jersey, New Brunswick, NJ, United States

**Keywords:** phenology, survival, development, climate, temperature

## Abstract

Spotted lanternfly (*Lycorma delicatula* White) is an invasive planthopper that was introduced to the United States from Asia and readily spreads *via* human aided means. Three geographically separated populations in the United States (NJ, PA, and WV) were collected and used to assess the effects of fluctuating thermal regimes that included temperatures above or below the upper (T_max_) and lower (T_min_) developmental thresholds, respectively, on nymphal survival and development, and to determine if there was within- and among-population variation in hatch timing and temperature responses of nymphs. Nymphs exposed to temperatures > T_max_ and <T_min_ were able to develop when those temperatures were part of an alternating regime, even though development took longer, and the average survival was lower than that of the corresponding constant temperature. When individuals from different geographically separated populations were exposed to the same temperature regimes, there was intra- and inter-population variation in time to hatch, instar duration, and estimated T_min_ values. The NJ population on average hatched earlier than the PA populations. There was 1-4°C difference in estimates of the T_min_ for the first through third instars for individuals from different populations. In addition, the time in instar estimates for constant 15 and 25°C from this study were 26 and 7 days faster, respectively, than estimates from previous studies. The variability in thermal responses documented in this study is large enough to have impacts on predicted phenology and potential risk of establishment especially in areas previously considered too cold to be at risk. This new information should be incorporated into phenology and risk models to improve their predictive ability.

## 1 Introduction

Extreme temperatures, close to or exceeding thermal thresholds, increase mortality and limit development along the climatic edges of a species geographic niche and can, in part, determine the potential distribution of invasive species in novel habitats. Invasive insect species with broader geographic ranges generally are assumed to have a wider thermal tolerance and/or more variation in performance tolerances among populations (1). The variation in thermal tolerances among populations can have a genetic basis, with differential selection occurring in local environments. Variation can also be the result of phenotypic plasticity, or the consequence of maternal or epigenetic effects, or a combination of these factors (1).

Inadvertent human-aided introduction (or spread) of species can rapidly create disconnected populations that are exposed to widely varying thermal environments. These environments can vary in the timing and severity of temperature extremes, number of days temperatures exceed the lower developmental threshold and variation around the mean annual temperature (2). Populations introduced to these novel environments may rely on thermal response variation present in the founding populations to allow establishment, and selective pressures may result in genetic divergence. For example, *Bemisia tabaci* Gennadius (Hemiptera: Aleyrodidae) and some cereal aphids, are invasive insects that have rapidly responded to thermal selection for heat tolerance which has allowed them to invade even tropical environments (3, 4). There are also cases of invasive insects developing higher tolerances to extreme cold and exhibiting variation among populations in cold tolerance, even a parthenogenetic species, *Adelges tsugae* Annand (Hemiptera: Adelgidae) (5). Other experiments using *Lymantria dispar* L. (Lepidoptera: Erebidae) have done simulated reciprocal transplants to assess the ability of different populations to deal with the northern and southern temperature extremes of the insect’s range, finding some populations outperform others in the new environments (6). Studying geographically distinct populations of an invasive species that is readily spread by human activity provides unique opportunities to assess its ability to utilize novel environments, look for population level variation in thermal responses, and assess where the species may be able to establish.

The spotted lanternfly (SLF) (*Lycorma delicatula* White [Hemiptera: Fulgoridae]) is an invasive planthopper that was introduced to the United States from Asia that readily spreads *via* human activity. It was first detected in Berks County, PA in 2014 and has since spread and established across the eastern United States (7, 8). In the first five years since its introduction, long distance dispersal events of up to 92 km have been documented (9). Primarily these long-distance movements occur when eggs are laid on vehicles (trains, cars, etc.) or materials that are stored outdoors and moved (8). This insect is a phloem feeder that utilizes over 100 hosts plants and causes direct or indirect damage to some economically important hosts as well as causing nuisance problems (10, 11). If unmanaged it is expected to spread throughout the United States to all suitable habitats by 2037 (12).

Using the modelling program MAXENT to estimate suitability in the U.S. from relationships of environmental variables at known occurrences in the native range, one study concluded the northernmost areas of New England, and the far southeastern US would be unsuitable for SLF establishment (13). Using a process-based modeling approach to determine spread probability over time, Jones et al. (12) showed that similar areas of both the northern and southern US may be at a low risk for spread and establishment. Whether SLF will be able to establish and spread as predicted by these models will be validated or disproven as the insect continues to invade new areas.

Previous studies focusing on the temperature responses of SLF have shown that this species can survive and develop at constant temperatures between 15 and 30°C (14). At constant temperatures outside this range, the species can survive for some time (2-35 days depending on temperature) but is unable to complete development (14). Lower developmental thresholds are estimated to be about 13°C for first and second instar nymphs and 6-8°C for third and fourth instar nymphs (14). Upper developmental thresholds are estimated to be 43°C for first instar nymphs and close to 35°C for all other instars (14). All this previous work has been done at constant temperatures in a laboratory using populations of SLF collected in Pennsylvania near the original introduction site and no work has been done to quantify the variation in temperature responses of the species throughout the entire range it now occupies or assess the actual thermal performance of the species in a more ecologically relevant way. In addition, a modeling effort to map the life-history of SLF in occupied and uninvaded ranges using all available laboratory temperature response data (15) had to adjust the developmental rate estimates by Kreitman et al. (14) to better match those in the field. This indicates there is a need for additional work on temperature responses of this species. Also, further work utilizing SLF thermal responses to model potential range in the invaded areas is needed.

No prior studies have assessed the effects of fluctuating temperature regimes on egg hatch nor survival or development of spotted lanternfly nymphs. Fluctuating temperatures within the permissive range (upper developmental threshold [T_max_] ≥ x ≥ lower developmental threshold [T_min_]) can improve insect performance, and in regimes that include stressful temperatures the permissive temperature portions can allow the insect to recover from the harmful effects of thermal extremes (temperatures outside the permissive range) (16). As a consequence, fluctuating regimes could allow development outside the permissive range, although development may be delayed, and high fluctuation amplitudes can cause more severe negative effects (16). Temperatures exceeding the estimated developmental thresholds of SLF nymphs occur in more northern and southern parts of the eastern US and at higher elevations where they may end up due to human aided transport. Understanding how extreme temperatures may affect this insect when part of normal daily fluctuations in temperature would improve phenology models and predictions of potential range.

There were two goals of this study. First, we assessed the effects of alternating regimes on SLF nymphal survival and development, using temperature exposures above and below the known developmental thresholds. Second, we determined if there was variation in hatch timing and temperature responses of nymphs from different SLF populations. Then we discuss how this information could impact and be incorporated into estimates of the SLF’s potential geographic range and phenology models.

## 2 Materials and methods

### 2.1 Source populations

One hundred and sixteen egg masses were collected October 20, 2020 from *Betula* sp. or dead trees along the bird watching path at Spruce Run Reservoir in Clinton, NJ (40° 39’47.03”N, 74°55’36.02”W), which is in the USDA Plant Hardiness Zone (https://planthardiness.ars.usda.gov/) 6A. On October 22, 2020, 135 egg mases were collected from trees (*Prunus serotina* Ehrhart [Rosales: Rosaceae], *Acer platanoides* L. [Sapindales: Sapindaceae], *Morus papyrifera* L. [Rosales: Moraceae] or *Crataegus monogyna* Jacquin [Rosales: Rosaceae]) in The Woodlands cemetery in Philadelphia, PA (PA1: 39° 56’45.86”N, 75°12’19.37”W) and 118 egg masses from trees (*Betula nigra* L. [Fagales: Betulaceae], *B. lenta* L. [Fagales: Betulaceae], *Acer rubrum* L. [Sapindales: Sapindaceae], *P. serotina*, or *Pinus strobus* L. [Pinales: Pinaceae]) in the Neshaminy State Park, Bensalem, PA (PA2: 40° 4’31.87”N, 75°55’0.59”W) which are both in the 7B plant hardiness zone. On January 15, 2021, 73 egg masses were collected from the bark of dead *Ailanthus altissima* (Miller) Swingle (tree of heaven [TOH]) (Sapindales: Simaroubaceae) trees in forest strips surrounding an industrial area in Winchester, VA (39°12’35.6”N, 78°11’18.7”W). To collect egg masses, we carefully chiseled through the bark around the egg mass and then lifted the bark off the tree without bending it.

### 2.2 Egg mass preparation and temperature treatments

Eggs collected in Winchester, VA were stored in a nearby barn (39°12’06.2”N 78°09’12.5”W, 6B plant hardiness zone) from the date of collection until March 10, 2021, when they were shipped over-night to the Forest Service Quarantine Laboratory in Ansonia, Connecticut. The Clinton, NJ and two PA populations were collected within a few weeks of being laid and brought directly back to the quarantine facility in CT. The two PA populations were combined (i.e., put together in cages) for all but the first instar treatments so the populations are referred to by the two-letter state for all nymphal data. The egg masses were brought into the quarantine laboratory and put on screens under a laminar flow hood for 30 minutes to remove excess moisture before being placed individually in 60 × 15 mm petri dishes (Corning Inc., Falcon product #351007). The petri dishes with egg masses were then held in clear plastic boxes (60-100 petri-dishes per box) and placed in either a chamber set to a constant 15°C, 65% RH and a 14:10 L:D cycle, or placed in a chamber in which the temperature cycled between the mean high and low temperatures for the specific week of the year (following a sign wave shape). The high and low temperatures, humidity, and light cycle was changed weekly to mimic the average weekly parameters in Napa, CA from 2010-2020 as reported by the National Oceanic and Atmospheric Administration (https://www.noaa.gov/). Napa was chosen for use as a validation data set for another study that is developing the phenology model and since it is a major grape growing region that is concerned about SLF establishment. In this study the Napa regime only served as a fluctuating regime that was closer to natural for hatching part of the egg masses. The 15°C regime was used because previous work has shown that the eggs will progress to hatch without lower temperatures, and this could provide information about how eggs would respond in areas where winters are mild. Forty-five egg masses each from the NJ and the two PA sites were place in the alternating regime; all the other egg masses were held at 15°C. The two temperature regimes (constant and variable) provided staggered hatch times which allowed greater repetition of cages in smaller growth chambers. Hatch was checked daily, and nymphs were removed for use in the study. Cumulative percent hatch for both populations, and for individual egg masses was tracked for the NJ population and two PA populations for eggs held at 15°C. The VA population could not be directly compared because it was overwintered at naturally occurring temperatures. All SLF egg masses were transported to Ansonia, CT where the Forest Service quarantine laboratory is located under Animal Plant Health Inspection and Pennsylvania State permits. Voucher specimens were deposited at the Yale Peabody Museum of Natural History, New Haven, CT.

### 2.3 Hosts

Spotted lanternfly nymphs were reared in caged enclosures containing both *TOH* and *Vitis labrusca* L. (Vitales: Vitaceae) (concord grape [grape]) vines as food sources, with one exception (see 1.5 for details). Mixed hosts were used since pervious work has shown they develop and survive better than when only offered single hosts (17).

*Ailanthus altissima* seedlings were grown in a greenhouse from locally (southern Connecticut) sourced seeds that had been stratified for more than one year at 4°C. Seeds were sprouted in Jiffy peat plugs (4 cm diameter), then potted in tree pots measuring 7.6 × 7.6 × 20.3 cm (CN-SS-TP-308, Greenhouse Megastore, Danville, IL). As trees grew, they were repotted into 16.5 (diam) x 17.8 (tall) cm black pots and then 22.2 (diam) x 27.3 (tall) cm black pots to support larger trees for use in the larger cages used for group rearing nymphs to specific instars (see section 2.3 for details). Through the rest of the paper the pots will be referred to as “tree pots” (7.6 cm a side) and “black pots” for the bigger pots (16.5 cm diam). Trees used for the larger group rearing cages were ~120 cm tall (including the pot) and had stems that were ≥ 1 cm diameter at the base. Trees used in treatment cages were ~77 cm tall (including the tree pot). The shorter trees were placed in two groups based on diameter, with 4-6 mm trees used in cages with first and second instars, and trees with diameters between 7-10 mm used in cages with third and fourth instars.

*Grape* vines were purchased from Double A Vineyard (Fredonia, NY) as bare root stock and received in Spring 2020. The vines were planted either one to a tree pot or 3-4 in the larger black pots, the same type of pots as used for the TOH seedlings. Single vines in tree pots with a minimum of one cane that was 1 m long were used in the treatment cages and for rearing first and second instar nymphs in rearing cages. The black pots with multiple vines were used in the larger rearing cages for third instar nymphs.

Prior to the addition of SLF nymphs, the soil in each pot was covered with a white paper towel, which was cut to allow the stems to pass through. Paper towels allowed water to pass to the soil and allowed gas exchange while preventing the insects from accessing the soil. The pots containing the trees and vines were fertilized monthly using Osmocote fertilizer (ICL Specialty Fertilizers, Summerville, SC) and watered daily or as needed to maintain soil moisture.

### 2.4 Rearing spotted lanternfly nymphs for instar-specific experiments

SLF nymphs were either placed directly into treatments right after hatch (first instars) or reared to the beginning of an instar (second – fourth) and then exposed to the treatments until the molt to the next instar (or death) occurred. Nymphs from each population and instar combination were reared in separate cages. Two sizes of cages were used: small 32.5 × 32.5 × 77.0 cm (BugDorm 4S3074) and large 60 x 60 x 120 cm (BugDorm 6S620) mesh cages with clear front and back (MegaView Science Co., Ltd, Taichung, Taiwan). The majority of the rearing cages were held in chambers set at 25°C, 65% RH and a 14:10 Light : Dark cycle but a few were held at room temperature (20-22°C) to slow development when necessary because of chamber space limitations. Initially one grape and two TOH pots were placed in each rearing cage, sized appropriately for the instar being reared (i.e., smaller plants for younger instars). First instars were reared in groups of 50-100 in small cages or 300-500 in large cages. Fifty to seventy second instars were reared in small cages and in groups of 150-350 in large cages. All third instars were reared in groups of 35-240 (higher numbers when held at room temperature) in large cages. Fresh plants were added weekly or more often if needed. Cages were checked daily and all new molts, molt skins, and dead nymphs were removed. Nymphs from different rearing cages from the same population and instar were combined to make the cohorts that were placed in the treatment cages.

### 2.5 Study treatments

#### 2.5.1 First and second instar treatments

**Figure 1:**
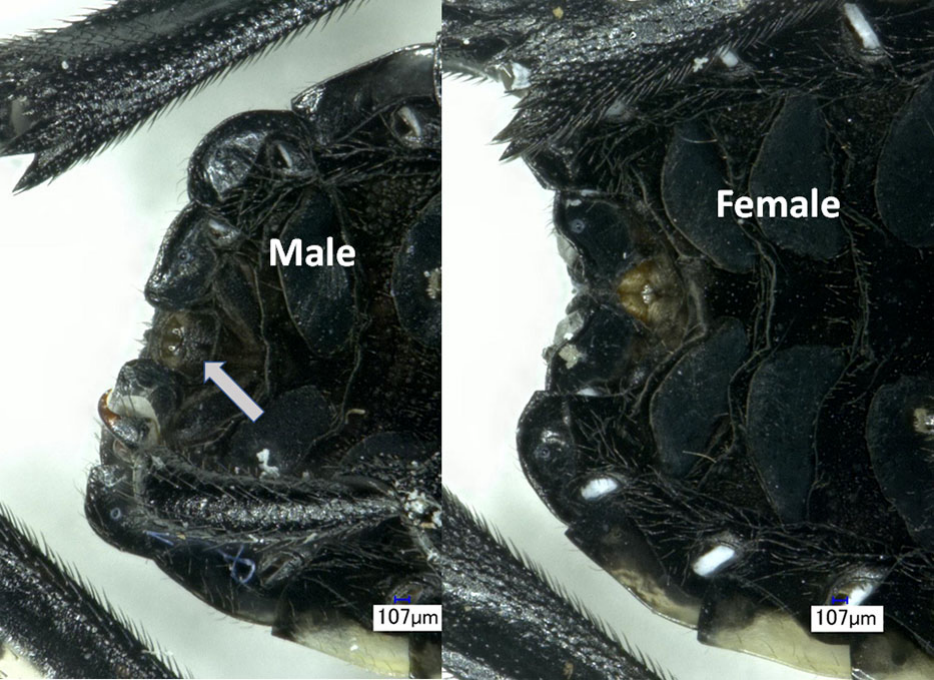
Differences between male (left) and female (right) SLF fourth instar nymph. Terminal ventral segments are shown with an arrow indicating the black heavily sclerotized band at the anterior end of the genital opening in the male, that is absent in the female.

First and second instar nymphs were exposed to three temperature treatments: constant 15°C, 8 hours at 5°C and 16 hours at 20°C, and constant 25°C. The first two treatments both provided an average daily temperature of 15°C, are consistent with temperatures that nymphs may experience in April and could be directly compared to assess the effects of the alternating regime. The temperature in the alternating regime was instantaneously switched by either modifying the temperature in the same chamber where the rearing cages were located (with the temperature being reached within<5 minutes, or by moving the cages containing the nymphs to another chamber already set with the next temperature. The 5°C portion of the alternating cycle exposed the nymphs to a temperature below the estimated developmental threshold (13°C for firsts and 12°C for seconds) (14) and the average low monthly temperature at the NJ site in April (https://www.worldweatheronline.com). This would be similar to a rapid drop in temperature during a spring cold wave that first and second instar nymphs might be exposed to. The two constant temperature treatments (constant 15 and 25°C) provided two points on a temperature response curve for each instar that could be used to see if different geographic populations responded the same way over this temperature range. Two cages with 30 first instar nymphs from each population (one cage from each location in PA) were set up in the first two treatments but only 20 per cage were used in the 25°C treatment cages (**Table 1**). The first instar nymphs in each cage came from multiple egg masses (6-16 egg masses for the PA and NJ populations, and 3-5 egg masses for the VA population). Three cages of second instars were set up for the two constant temperatures, 20 nymphs per 15°C cage and 10 nymphs per 25°C cage. A total of four cages of 20 second instar nymphs were set up in the alternating regime, 3 cages from the VA and 1 cage form the NJ population. This was because the chamber space for the alternating regime was limited and the timing of the seconds from the PA populations did not coincide with when space was available. Daily counts were made of the dead and newly molted nymphs, which were removed from the cages. All newly molted nymphs were weighed and frozen. New third instars were sexed by looking at the terminal ventral segments (**Figure 1** shown for fourth instar nymphs but thirds are the same just smaller). Males have a black heavily sclerotized band at the anterior end of the genital opening that is absent in the female. Time in instar was calculated for each nymph. Any nymphs that drowned, were consumed by a spider (spiders were occasionally found due to greenhouse exposure to host plants) or were otherwise accidently killed were censored from the data before percentage mortality was calculated.

**Table 1:** Summary of experimental design for nymphal (cages/number of nymphs in each cage) work.

Temperature Treatment (°C)	Population	Instar			
		First	Second	Third	Fourth
**15**	NJ	2 cages/30 nymphs	3 cages/20 nymphs	3 cages/15 nymphs	
	PA	2 cages/30 nymphs	3 cages/20 nymphs	3 cages/15 nymphs	
	VA	2 cages/30 nymphs	3 cages/20 nymphs	4 cages/15 nymphs	
**20/5**	NJ	2 cages/30 nymphs	1 cages/20 nymphs		
	PA	2 cages/30 nymphs			
	VA	2 cages/30 nymphs	3 cages/20 nymphs		
**25**	NJ	2 cages/20 nymphs	3 cages/10 nymphs	4 cages/15 nymphs	5 cages/10 nymphs
	PA	2 cages/20 nymphs	3 cages/10 nymphs	5 cages/15 nymphs	4 cages/10 nymphs
	VA	2 cages/20 nymphs	3 cages/10 nymphs	5 cages/15 nymphs	2 cages/10 nymphs
**35/20**	NJ			4 cages/15 nymphs	3 cages/10 nymphs
	PA			5 cages/15 nymphs	3 cages/10 nymphs
	VA			4 cages/15 nymphs	2 cages/10 nymphs
**40/20**	PA			2 cages/15 nymphs	
	VA			2 cages/15 nymphs	

Temperature treatments: 20/5 = 8 hours at 5°C and 16 hours at 20°C, 35/20 = 35°C for 8 hours and 20°C for 16 hours, and 40/20 = 40°C and 20°C for 6 and 18 hours.

#### 2.5.2 Third and forth instar treatments

Third instar nymphs were exposed to four temperature treatments: Two constant temperature regimes (15°C and 25°C) and two fluctuating regimes. One fluctuating regime exposed the nymphs to 35°C for 8 hours and 20°C for 16 hours (for an average temperature exposure of 25 degrees C), and 40°C and 20°C for 6 and 18 hours, respectively, yielding an average temperature of 25 degrees. Fourth instars were exposed to only the 25°C constant and the 8 hours at 35°C and 16 hours at 20°C treatments because of insufficient chamber space and limited numbers of surviving fourth instar nymphs for use in the study. Temperature changes were handled here the same way they were handled in the first and second-instar nymph studies described above. The two alternating temperature treatments provided an average daily temperature of 25°C which is the average monthly temperature in July for many areas where SLF is found. 35°C represents the estimated upper developmental threshold for the third and fourth instars and the 40°C part is above the threshold (14). These treatments are meant to approximate what third and fourth instars might be exposed to during a short heat wave during June or July in areas where the SLF is found or could potentially disperse into. The two constant temperatures also allowed between population comparisons of responses to temperatures. A total of 7 cages at 15°C (3 NJ, 3 PA, and 4 VA), 14 cages at 25°C (4 NJ, 5 PA, and 5 VA), 13 cages at 35/20°C (4 NJ, 5 PA, and 4 VA), and 4 cages at 4/20°C (2 PA, and 2 VA) of third instar nymphs were setup with an average of 15 nymphs per cage (range 6-20) (**Table 1**). Fewer fourth instar cages averaging 10 per cage (range 6-11) were setup as follows: 11 cages at 25°C (5 NJ, 4 PA, and 2 VA), 8 cages at 35/20°C (3 NJ, 3 PA, and 2 VA). All newly molted individuals were weighed, frozen, and sexed. Time in instar was calculated for each nymph. Nymphs were censored from the data in the same way as detailed for first and second instars before percentage mortality was calculated.

#### 2.5.3 Cages and hosts

All treatments for all instars were conducted in cages which were 32.5 × 32.5 × 77.0 cm (BugDorm 4S3074). Initially, each cage had one TOH tree and one grape vine placed in them. An exception to this was applied to the first and second instar cages held at a constant 25°C, which included only TOH (no vines in 2 first and 3 second instar PA cages and 2 first and 1 second NJ cages) as these cages were used simultaneously in a related study on host plants (Kreitman et al. in press this journal) that only used one host per cage. Overlap in the studies was necessary since nymphs and space were both limited and the imbalance in sample structure is addressed in the methods and results.

The TOH plants were replaced every 21, 21, 7, 4, and 4 days in the constant 15°C, alternating 5/20°C, constant 25°C, alternating 35/20°C, and alternating 40/20°C trials. The differences in the rotation time for plants allowed for the maintenance of host quality under conditions where nymphs were developing and depleting hosts more rapidly. Grape vines were not replaced.

### 2.6 Statistical analysis

Kaplan-Meier product limit estimates of the survival functions were used to calculate the days with 95% confidence intervals that 90, 75 and 50 percentiles of nymphs survived when exposed to different temperature treatments (18). This method of estimating survival functions was used because it can handle the censored data (individuals that survive and molt) and does not require any assumptions about the shape of the function. Nymphs that drowned, were consumed by spiders, or accidentally killed were removed from the analysis since their time of death was unnatural. A Mantel-Haenzel test was used to compare the survival between two temperature treatments so that an adjustment could be made for the potentially confounding factor of differences between populations in survival (18).

The normality of the data was checked using a Shapiro-Wilk test and when the data was not normally distributed was right-skewed, a PROC UNIVARITE was used to assess the fit of a gamma distribution (19). Time (days) to hatch at 15°C, time (days) in instar and newly molted nymphal weights (mg) were analyzed using PROC GLIMMIX (19). The time in instar data was fit to a gamma distribution with a log link, and the hatch and weight data were fitted to a normal distribution. Residuals analyses using Levene’s test indicated that variances were equal. Models that compared multiple temperature treatments accounted for population differences by including population as a random effect, and models that compared populations within a single temperature treatment included cage as a random effect. Models for data obtained for first instars (i.e. time spent as a first instar and weight of newly molted second instars) had temperature treatment as a fixed effect and all other models (i.e. those involving instars 2-4 for time and 3-4 for weight) had sex and the interaction between temperature treatment and sex added as fixed effects. The model for time to hatch just had population as a fixed effect. Differences between means were assessed using the Tukey-Kramer test with an α = 0.05 (19).

Rough estimates (based on only two temperatures) of the lower threshold for development (T_min_) were calculated using the constant temperature data (individual values) for each instar and population. First the relationship between temperature (t) and developmental rate (y = 1/time in instar (days)) was fit to a linear model using Excel (Microsoft Corporation software) following:


y = bt + a

Then, the intercept (a) was divided by the slope (b) to calculate T_min_ (the x-intercept). Estimates for *a* and *b* were calculated by using least squares regression (18). The population regression lines were then compared for equality of variance (Bartlett’s test (20)), if variances were equal then slopes were compared, and if slopes were also equal then the y-intercepts of the lines were compared to determine if the lines were the same or not (18). The standard error on the T_min_ estimate was calculated using the method developed by Campbell et al. (21).

## 3 Results

### 3.1 Nymphal survival

**Figure 2:**
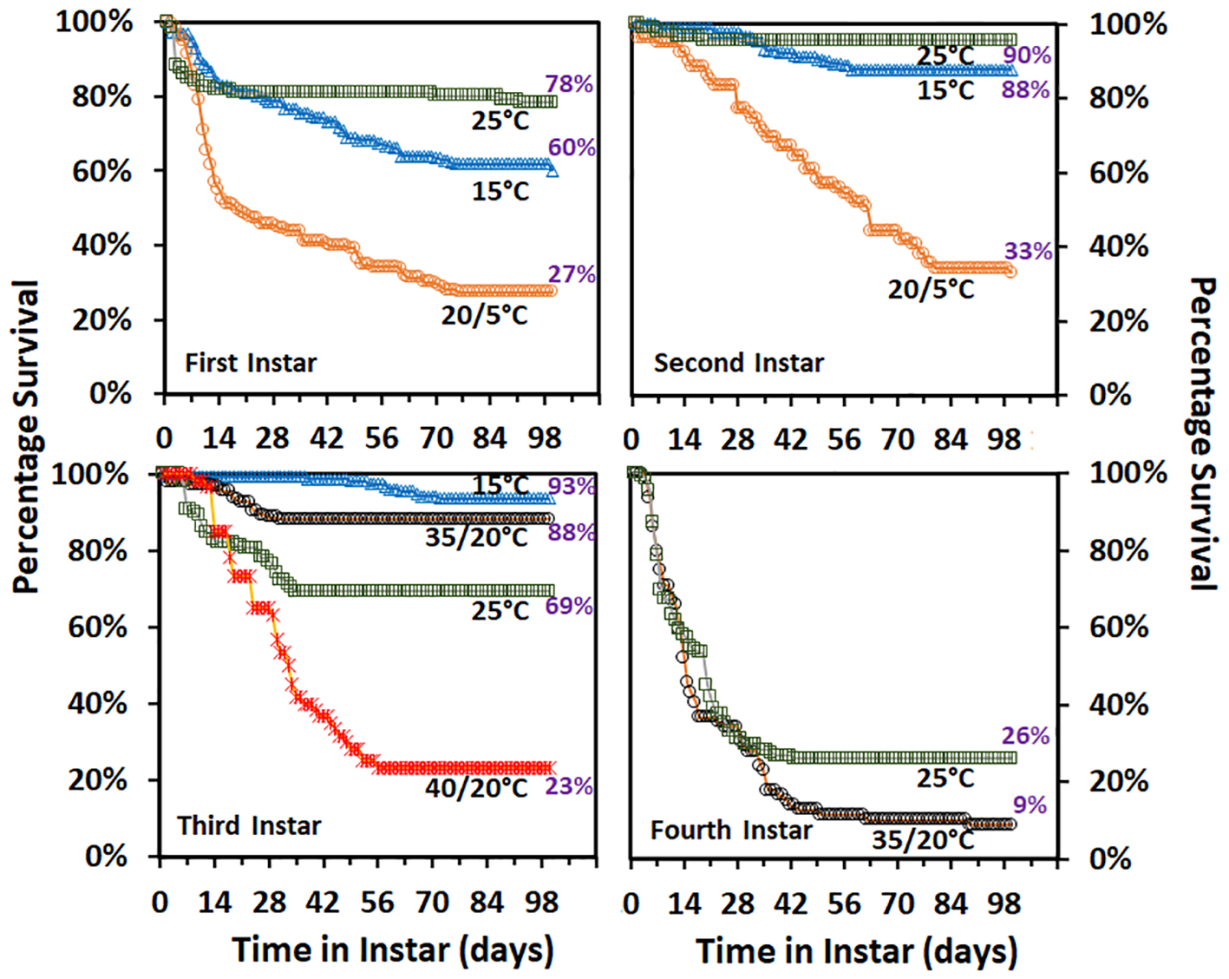
Percentage survival of nymphal SLF held in different temperature treatments by instar. Total percentage nymphs that molted is shown at 100 d. Temperature treatments: 20/5 = 8 hours at 5°C and 16 hours at 20°C, 35/20 = 35°C for 8 hours and 20°C for 16 hours, and 40/20 = 40°C and 20°C for 6 and 18 hours.

Percentage survival curves for each temperature treatment and instar are provided in **Figure 2**. About half of the decline in first instar survival for the 15°C and 20/5°C treatments occurred in the first 14 days and then losses of nymphs in both treatments dropped to a rate of about 1% every five days until about day 77. Survival in the 20/5°C treatment was almost half that of the 15°C treatment despite the average temperature being the same. Most of the declines in survival for the 25°C treatment occurred in the first 14 days and only a few individuals that did not molt survived until about 70 days then died.

Second instar nymph survival declined at a constant rate of about 5% every 5 days for the first 77 days in the 20/5°C treatment while survival of nymphs at 15°C was high during the first 28 days and then declined at a rate of 1% every three days for the next 28 days. Ninety-six percent of second instar nymphs at 25°C survived and all the declines in survival occurred in the first 19 days.

Declines in survival of third instar nymphs began at about 7 days in the 25, 35/20, and 40/20°C treatments but did not start until about 35 days in the 15°C treatment. No declines in survival occurred after 28, 35, 56, and 72 days for the 35/20, 25, 40/20, and 15°C treatments respectively. The nymphal survival in the 40/20°C treatment was less than half that in the 25°C treatment which was less than half that of the 35/20°C treatment. Fourth instar survival was low in both temperature treatments, but lower in the 35/20°C treatment than the 25°C treatment. The sharpest declines in survival occurred in the first 42 days in both treatments.

Estimates of the days that 90, 75 and 50 percentiles of nymphs survived in each treatment and instar combination are provided in **Table 2**. These estimates predicted that 90% of nymphs will survive 7-15 long cold snaps and 6-to-24-day heat waves, depending on the instar and intensity of the temperature extreme. The observed nymphal survival was different for all within instar temperature treatment comparisons, adjusted for population differences, where treatments had the same average daily temperature (**Table 3**).

**Table 2:** The 90, 75 and 50 percentile estimates of the number of days (95% confidence intervals) SLF nymphs in each instar survived in each temperature treatment.

Temperature Treatment (°C)	Estimated Survival Percentile	Instar				
		First	Second	Third	Fourth
**15**	**90**	9.0 (8-13)	43.6 (33-56)	65.0 (54-72)	
	**75**	37.8 (18-47)	57.1 (50-NA)	71.7 (62-NA)	
	**50**	60.9 (53-74)	NA	NA	
**20/5**	**90**	7.0 (5-8)	15.0 (2-22)		
	**75**	10.0 (9-11)	29.5 (21-43)		
	**50**	15.0 (13-25)	55.7 (43-63)		
**25**	**90**	3.0 (3-8)	18.1 (12-NA)	8.2 (7-11)	6.0 (6-7)
	**75**	16.9 (10-87)	NA	25.5 (14-28)	8.0 (7-9)
	**50**	NA	NA	29.0 (26-31)	20.0 (14-24)
**35/20**	**90**			24.0 (19-28)	6.0 (5-7)
	**75**			30.3 (24-NA)	8.0 (7-12)
	**50**			NA	15.0 (13-18)
**40/20**	**90**			14.0 (12-18)	
	**75**			19.0 (14-29)	
	**50**			32.5 (29-37)	

Estimates were obtained using the Kaplan-Meier product limit estimates of the survival functions (18). Percentiles that could not be calculated because mortality was low in that treatment are denoted as NA (not available). Cells in the table that are greyed out are treatment and instar combinations that were not done. Temperature treatments: 20/5 = 8 hours at 5°C and 16 hours at 20°C, 35/20 = 35°C for 8 hours and 20°C for 16 hours, and 40/20 = 40°C and 20°C for 6 and 18 hours.

**Table 3:** Comparisons of the survival between pairs of temperature treatments for nymphs of SLF reared using the same average daily temperature exposure, adjusted for population differences in survival.

Instar	Treatment 1	Treatment 2	Statistics		
			Chi Squared	Degrees of freedom	P value
**First**	**15°C**	**20/5°C**	37.2	1	< 0.0001
**Second**	**15°C**	**20/5°C**	54.9	1	< 0.0001
**Third**	**25°C**	**35/20°C**	20.0	1	< 0.0001
**Third**	**25°C**	**40/20°C**	43.5	1	< 0.0001
**Third**	**35/20°C**	**40/20°C**	75.2	1	< 0.0001
**Fourth**	**25°C**	**35/20°C**	9.3	1	0.0023

Statistics are for a Mantel-Haenzel test with population (from NJ, PA, and VA) as a random effect (18). Temperature treatments: 20/5 = 8 hours at 5°C and 16 hours at 20°C, 35/20 = 35°C for 8 hours and 20°C for 16 hours, and 40/20 = 40°C and 20°C for 6 and 18 hours.

### 3.2 Impact of extreme temperature alternations on instar duration

The first instar was significantly longer (6 d) when nymphs were exposed to the 20/5°C treatment than the constant 15°C treatment (F = 21.08, df = 1. 15, p<0.0001, **Table 4**). Duration of the second instar did not differ by temperature treatment (F = 0.99; df = 1, 166; p =0.32), sex (F = 0.63; df = 1, 166; p =0.43) or the interaction between the two (F = 0.04; df = 1, 166; p =0.84). The time in the third instar differed between temperature treatments (F = 79.62; df = 2, 302; p =0.00) and with sex (F = 11.83; df = 1, 302; p =0.00), but not the interaction between the two (F = 1.13; df = 2, 302; p =0.32). The third instar was the shortest (male 17d and female 19 d) when nymphs were exposed to constant 25°C, longer (male 20 d and female 23 d) when exposed to 35/20°C, and longest (male 28d and female 33 d) when exposed to 40/20°C. Duration of the fourth instar varied with temperature (F = 8.56; df = 1, 35; p =0.01) but not with sex (F = 3.15; df = 1, 35; p =0.08), or the interaction between the two (F = 0.76; df = 1, 35; p =0.39). As with the third instars the fourth instar duration was longer (6 d in females and 10 d in males) for nymphs exposed to 35/20°C than those exposed to constant 25°C. All these comparisons treated population as a random effect to account for between population variation.

**Table 4:** Developmental time (days) and newly molted weights (mg) for different nymphal instars of SLF exposed to different temperature treatments (mean *+* SE (*n*)).

Instar	Sex	Temperature Treatment	Time in Instar (Days)^a^	Newly Molted Weight (mg)^a^
**First**	**U**	**15°C**	45.30 ± 3.02a (110)	6.98 ± 0.07a (110)
**First**	**U**	**20/5°C**	51.31 ± 3.53b (48)	6.68 ± 0.10b (48)
**Second**	**F**	**15°C**	43.66 ± 1.79a (66)	21.91 ± 0.62b (66)
**Second**	**F**	**20/5°C**	41.76 ± 3.01a (10)	28.15 ± 1.85a (10)
**Second**	**M**	**15°C**	42.34 ± 1.66a (90)	19.11 ± 0.48c (90)
**Second**	**M**	**20/5°C**	39.65 ± 3.47a (6)	18.45 ± 1.53bc (6)
**Third**	**F**	**25°C**	18.62 ± 2.11d (62)	55.11 ± 2.89ab (62)
**Third**	**F**	**35/20°C**	23.41 ± 2.65b (70)	55.70 ± 2.90a (70)
**Third**	**F**	**40/20°C**	32.63 ± 4.60a (4)	46.37 ± 3.61bc (4)
**Third**	**M**	**25°C**	17.14 ± 1.94d (78)	43.85 ± 2.27c (78)
**Third**	**M**	**35/20°C**	20.32 ± 2.29c (86)	43.80 ± 2.26c (86)
**Third**	**M**	**40/20°C**	27.93 ± 3.47a (10)	40.91 ± 2.57c (10)
**Fourth**	**F**	**25°C**	29.72 ± 2.05a (14)	142.30 ± 10.98a (14)
**Fourth**	**F**	**35/20°C**	35.80 ± 4.68a (20)	138.50 ± 13.12a (20)
**Fourth**	**M**	**25°C**	23.40 ± 1.42b (3)	103.90 ± 7.82b (3)
**Fourth**	**M**	**35/20°C**	33.05 ± 3.75a (4)	90.87 ± 8.06b (4)

a Within instars (across all temperature treatments and sexes), means followed by the same letter are not significantly different based on a Tukey test with α = 0.05 (19). Temperature treatments: 20/5 = 8 hours at 5°C and 16 hours at 20°C, 35/20 = 35°C for 8 hours and 20°C for 16 hours, and 40/20 = 40°C and 20°C for 6 and 18 hours.

### 3.3 Newly molted nymphal weights

Nymphs that molted to the second instar in the 20/5°C treatment weighed 0.3 mg more than those in the constant 15°C treatment (F = 5.81; df = 1, 154; p =0.02; **Table 4**). The weight of newly molted third instars did not differ by temperature treatment (F = 3.8; df = 1, 166; p =0.05), but did by sex (F = 27.52; df = 1, 166; p =0.00) and the interaction between the two (F = 7.17; df = 1, 166; p =0.01). Females that molted to the third instar in the 20/5°C treatment weighed 6.2 mg more than those in the 15°C treatment. The weight of nymphs that molted to the fourth instar differed between temperature treatments (F = 5.92; df = 2, 302; p =0.00) and with sex (F = 60.69; df = 1, 302; p =0.00), but not the interaction between the two (F = 1.25; df = 2, 302; p =0.29). Fourth instar females weighed the least when exposed to the 40/20°C treatment and weighed about the same as males in all three temperature treatments. Weights of individuals that molted to the adult only differed by sex (F = 69.44; df = 1, 35; p =0.00) and not temperature (F = 3.23; df = 1, 35; p =0.08) or the interaction between the two (F = 1.4; df = 1, 35; p =0.24). New adult females weighted more than males in both temperature treatments.

### 3.4 Between population variation in hatch timing and time in instars

**Figure 3:**
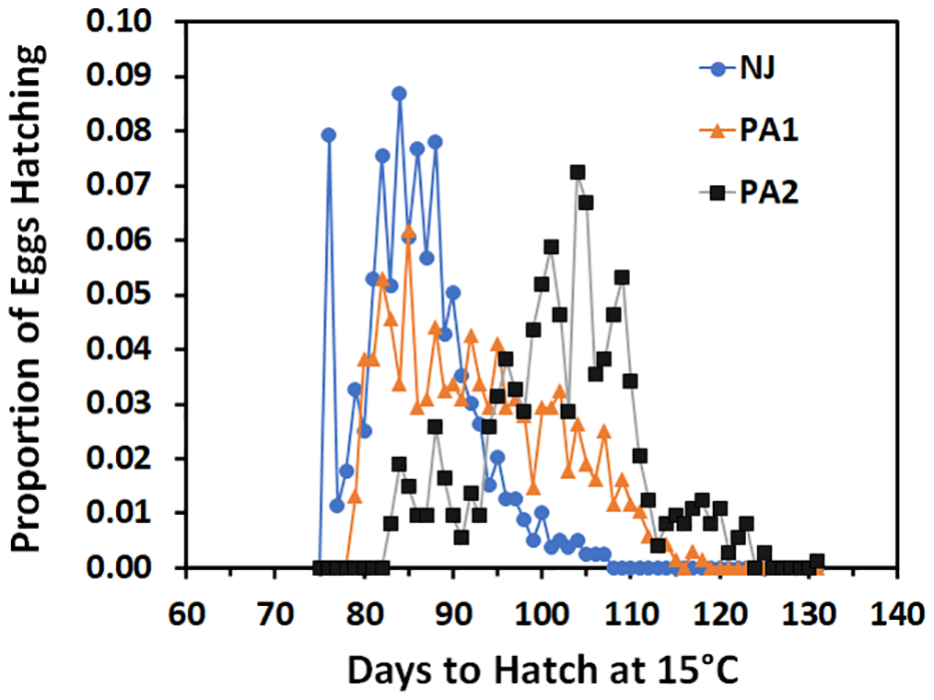
Proportion of SLF eggs from three different populations held at 15°C that hatched over time (days).

Time to hatch varied by population (F = 781.72; df = 2, 2205; p =0.00). The NJ eggs hatched faster (86.0 ± 0.3 days) than PA1 eggs (92.9 ± 0.3 days) which hatched faster than the PA2 eggs (102.2 ± 0.3 days) when held at 15°C. Although the average time to hatch differed between populations, there was substantial overlap in the hatch timing (**Figure 3**). The PA2 population appears to have three modes, one small mode roughly corresponding to the distribution of the NJ population (75-95 days), a large mode with a mean about 20 days later than the NJ mean (95-115 days) and a final smaller mode with a mean close to 120 days. The PA1 population hatch appears to span most of the range of the other two populations with possibly two modes falling at the mean of the NJ population and corresponding to the largest mode of the PA2 population. These modes resulted from differences between egg masses in mean time to hatch and the size of the mode corresponded to the percentage of egg masses with that hatch timing (**Table 5**). There was also evidence that the timing of when the first NJ individuals hatched was missed, most of the eggs that hatched for two eggs masses were found on day 76 when hatch for most egg masses was spread out over multiple days (duration of hatch averaged NJ 7.8 ± 2.7, PA1 7.7 ± 5.0, and PA2 9.3 ± 5.2 days).

**Table 5:** Proportions of SLF egg masses with mean time to hatch in each of three groups for three populations of spotted lanternfly.

	Percentage of Egg Masses with Mean Time to Hatch (days)		
Population	76-94	95-114	115-125
**NJ**	82.4	17.6	0.0
**PA1**	56.3	43.8	0.0
**PA2**	10.6	78.7	10.1

**Figure 4:**
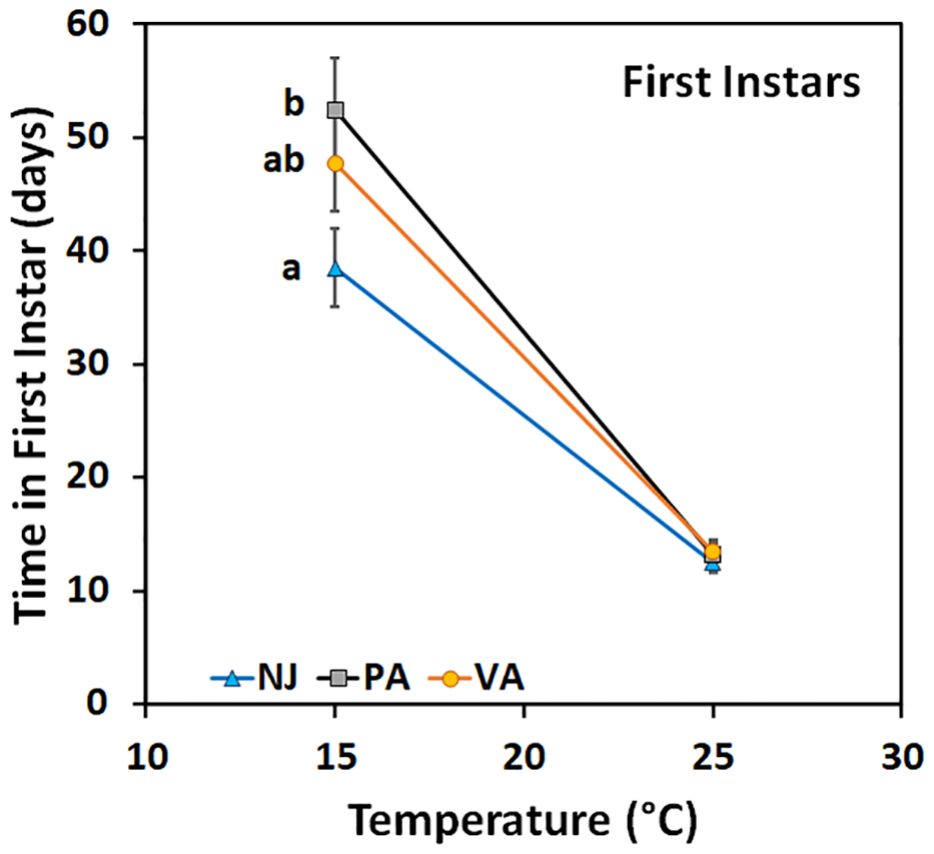
Time in instar (days ± SE) for first instar SLF from three populations held at two constant temperatures. Values within a constant temperature across both sexes followed by a different letter are statistically different (Tukey α< 0.05) and if no letter are shown there were no differences between populations.

**Figure 5:**
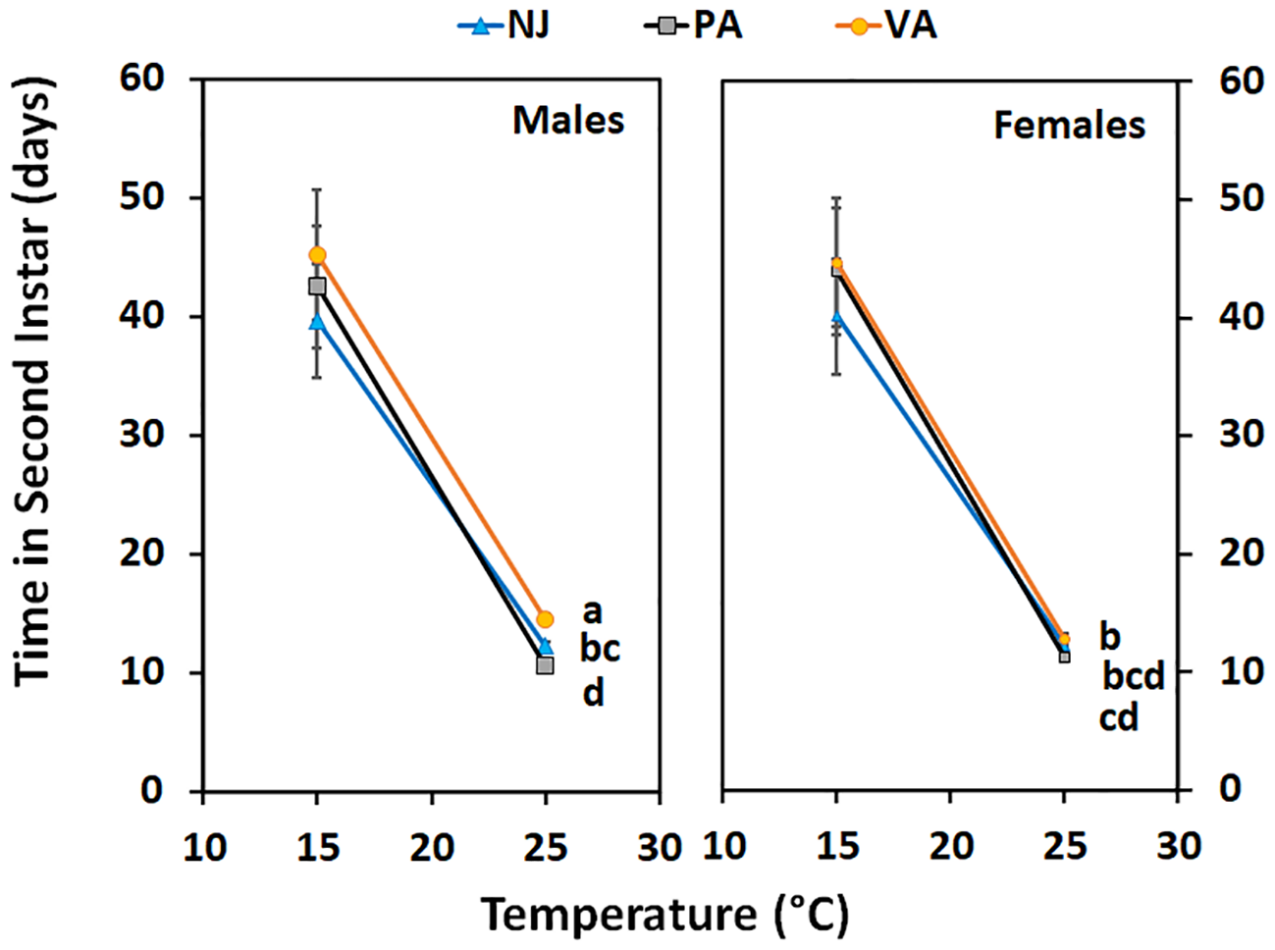
Time in instar (days ± SE) for second instar SLF of each sex from three populations held at two constant temperatures. Values within a constant temperature across both sexes followed by a different letter are statistically (Tukey α< 0.05) different and if no letter are shown there were no differences between populations.

Time in the first instar varied by population when nymphs were held at 15°C (F = 3.22; df = 2, 104; p =0.04) but not when held at 25°C (F = 0.3; df = 2, 88; p =0.74) (**Figure 4**). Nymphs from the NJ population completed the instar faster at 15°C than did those from the PA population, whereas the VA nymphs completed the instar with an intermediate number of days compared to the other populations. Time in the second instar for nymphs held at 15°C did not differ between populations (F = 0.26; df = 2, 144; p =0.77), by sex (F = 0.41; df = 1, 144; p =0.52), and there was no interaction between the two (F = 0.54; df = 2, 144; p =0.59). Nymphs of both sexes from the NJ population tended to complete the instar faster than those from the other populations which might impact phenology despite not being statistically significant. However, when second instars were held at 25°C the time in instar differed by population (F = 15.72; df = 2, 72; p =0.00) and the interaction between population and sex of the resulting third instars (F = 6.85; df = 2, 72; p =0.00), but not by sex (F = 1.32; df = 1, 72; p =0.26) (**Figure 5**). Male second instars from the PA population completed the instar faster at 25°C than those from the NJ population which completed the instar faster than those from the VA population. Female second instars from the PA population completed the instar faster than those from the VA population and those from the NJ population took an intermediate number of days to complete the instar at 25°C. The PA population had the only difference between sexes in the number of days in the second instar, with females completing it faster than males.

**Figure 6:**
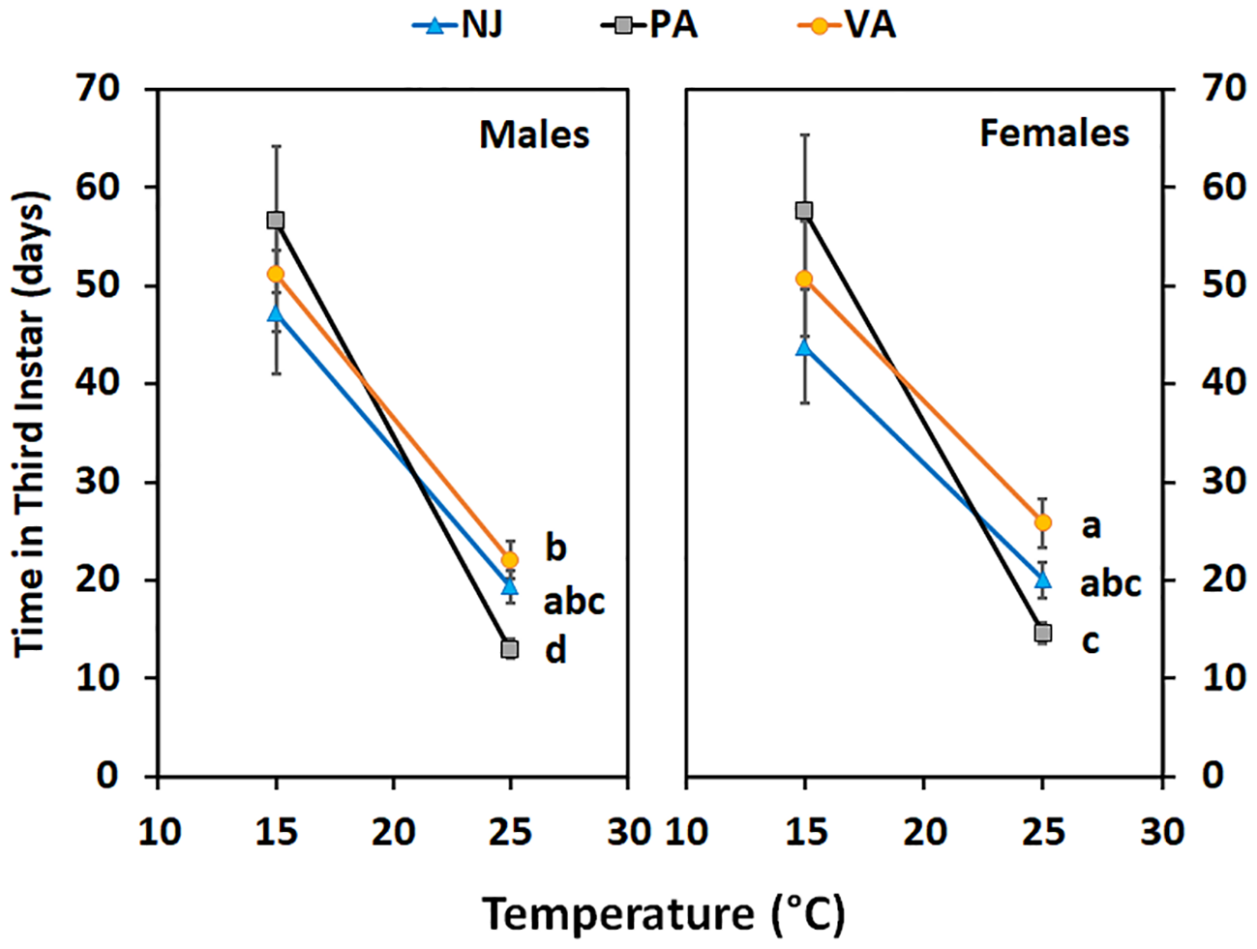
Time in instar (days ± SE) for third instar SLF of each sex from three populations held at two constant temperatures. Values within a constant temperature across both sexes followed by a different letter are statistically (Tukey α< 0.05) different and if no letter are shown there were no differences between populations.

Time in the third instar for nymphs held at 15°C did not differ between populations (F = 0.26; df = 2, 144; p =0.77), by sex (F = 0.41; df = 1, 144; p =0.52), or the interaction between the two (F = 0.54; df = 2, 144; p =0.59) (**Figure 6**). However, the trend was the same as seen at 15°C for the first instars; nymphs from the NJ population completed the instar faster than those from the PA population and the VA nymphs completed the instar in an intermediate number of days. When third instars were held at 25°C the time in instar differed by population (F = 12.32; df = 2, 124; p =0.00) and sex (F = 15.91; df = 1, 124; p =0.00), but not by the interaction between the two (F = 1.89; df = 2, 124; p =0.16). The time in third instar for both sexes for nymphs from the PA population was shorter than that of nymphs from the VA population and the NJ nymph time in instar was intermediate to that of the other two populations. Female nymphs from both the PA and VA populations spent longer in the third instar than did males from the same population.

### 3.5 Between population variation in estimated lower developmental thresholds

The parameters for the linear regressions for developmental rate verses temperature and the estimated T_min_ for each population and instar combination are given in **Table 6**. The T_min_ for the first instars varied by 1-2°C between populations with NJ (10.04°C) being the lowest and PA (11.59°C) being the highest. For second and third instar nymphs, the estimated T_min_ of the NJ and VA populations were similar while the PA population had a 1-4°C higher estimated value. The slopes of the first instar and second instar lies were significantly different. The assumption of equal variances was not valid for the third instar lines so comparisons of slope and Y-intercepts could not be done. Third instar variation increased with temperature.

**Table 6:** Parameters (± SE) for developmental rate verses temperature regressions and estimated lower developmental thresholds based on the 15 and 25°C data for each SLF population and nymphal instar combination.

Instar	Population	Slope	Intercept	Adj. r^2^	Estimated T_min_	Comparison of population lines^a^
**First**	**NJ**	0.00533 ± 0.00025	-0.05323 ± 0.00245	0.97	10.04 ± 0.21	Equal variances: χ^2^ = 4.24, df 2, p=0.1199 Equal slopes: F = 4.59; df 2, 198; p=**0.0112** Slopes are different
	**PA**	0.00587 ± 0.00153	-0.06841 ± 0.00305	0.96	11.59 ± 0.21	
	**VA**	0.00536 ± 0.00145	-0.05912 ± 0.00306	0.95	10.94± 0.26	
**Second**	**NJ**	0.00566 ± 0.00125	-0.05907 ± 0.00239	0.96	10.37± 0.18	Equal variances: χ^2^ = 5.82, df 2, p=0.0545 Equal slopes: F = 33.82; df 2, 236; p<**0.0001** Slopes are different
	**PA**	0.00667 ± 0.00132	-0.07628 ± 0.00257	0.97	11.39± 0.15	
	**VA**	0.00503 ± 0.00163	-0.05240 ± 0.00308	0.92	10.48± 0.27	
**Third**	**NJ**	0.00533 ± 0.00025	-0.05323 ± 0.00245	0.97	7.31± 0.68	Equal variances: χ^2^ = 8.06, df 2, **p=0.0178** Variances are different, assumptions for further comparisons not valid
	**PA**	0.00533 ± 0.00025	-0.05323 ± 0.00245	0.97	11.88± 0.34	
	**VA**	0.00533 ± 0.00025	-0.05323 ± 0.00245	0.97	8.07± 0.67	

Analyses were done using Statistix (18) and the sexes were combined.

The Bartlett’s test statistics on the comparison of the lines is provided.

In the comparison of population lines, the bold is the significant p-values.

## 4 Discussion

Several findings from this study could have an impact on the projected potential range of SLF and its ability to utilize novel habitats where human-aided transport takes it. Nymphs exposed to temperatures > T_max_ and <T_min_ were able to develop when those temperatures were part of an alternating regime with a favourable temperature, even though development was slower, and survival was lower than at the average corresponding constant temperature (**Table 4**). Additionally, when individuals from geographically distant populations were exposed to the same temperature regimes there was intra- and inter-population variation in time to hatch, instar duration, and estimated T_min_ values (**Tables 5**, **6**).

When insects are exposed to temperatures near their critical thermal minimums (the temperature at which locomotion stops, different from the T_min_), they enter a state called a chill-coma that is reversable, where coordinated movement does not occur (22). For the first few days when first instar nymphs were first moved to 5°C during the 20/5°C alternating regime they would fall off the plants and lay upside down as if dead for a few minutes them get up and return to the plants. This suggests that 5°C was cold enough to cause cold stress but that the insects were able to acclimate to it. Cold stress causes oxidative damage, decreased potential in neuromuscular membranes and disruption of the ion/water homeostasis across cell membranes, but exposure to warmer, favourable temperatures provide an opportunity for cells to effect repairs (23, 24). The cumulative effects of the stress still had negative effects on survival and delayed development. However, the negative effects were not as pronounced in the second instar, indicating that it may have a slightly different temperature tolerance. Sensitivity to temperature has been shown to vary independently across stages (25). In the SLF the first instar nymphs are the most likely to experience the cold temperatures, and they have a broader range of temperatures that they tolerate than do the second instars. The delay in development in the first instar was also only 6 days (**Table 4**) which indicates that some development must have occurred at 5°C. The first instar’s ability to survive in an alternating regime that includes 5°C suggests the estimated T_min_ reported in Kreitman et al. (14) may be inaccurate. So, it is likely that SLF would be able to survive and develop in colder environments than previously thought.

When insects are exposed to temperatures near T_max_ many potentially irreversible changes occur in insects that can lead to death or deleterious effects on biology and morphology: altered cellular pH and ion concentrations, changes in macromolecules (e. g. proteins, DNA, RNA, lipids), and alterations in cell structures (26). In addition, small increases in temperature can have increasingly stronger effects until abruptly hitting the lethal temperature (27). This fits with what was observed in this study. When third and fourth instar SLF were exposed to 35 or 40°C temperatures as part of an alternating regime with exposure to 20°C, development was delayed compared to the average constant temperature (25°C, **Table 4**). The increased time spent in the instar, especially in the 40/20°C regime, suggests that the nymphs were not able to develop or developed at a much slower rate during the extreme temperature part of the regime. Survival however declined dramatically for third instar nymphs as the amplitude of the difference between the temperatures increased: <20% mortality in 35/20°C and close to 80% in the 40/20°C regime (**Figure 2**). Fourth instar mortality was high in general due to the limitations of the laboratory rearing environment, but survival in the alternating regime was higher than the constant temperature regime. Consequently, SLF can develop when temperatures above T_max_ (estimated to be 35°C for third and fourth instar nymphs (14)) are part of an alternating regime just as has been observed for *Drosophila melanogaster* Meigen (Diptera : Drosophilidae) (16). Greater detrimental effects not only increase as the temperature increases but become more profound when the amplitude of the difference between high and low temperatures increases because of increasing energy demands. The percentage deviation between constant and alternating temperatures is generally smaller if the amplitude of the fluctuations is <7°C and larger if >7°C (28). If this holds true for SLF then as it moves south or into warmer regions it may reach thermal conditions that may limit its range but that the natural diurnal alternation of temperatures may buffer it somewhat from the deleterious effects. The benefits of alternating temperatures may however be minimal when close to the upper thermal limit, only extending the tolerable temperature range by ≤ 1°C (29). Care should be taken in extrapolating these results to the field, since the study used instantaneous changes in temperature whereas temperature in the field generally changes more gradually.

The average time spent in each instar when reared at 15 and 25°C was shorter in this study than reported by Kreitman et al. (14). First instars completed development at 15°C an average of 26 days faster and third and fourth instars completed development at 25°C an average of 7 days faster than previously reported. These differences are substantial and could affect the predicted phenology of SLF when used in a model. One SLF model that attempted to use the previously reported developmental rates had to adjust them to match developmental rates with those in the field (15) and reported for other hosts in the laboratory (30). The adjustments made for the modelling effort were to speed the developmental rate up for each instar, especially for the third and fourth instars (2.13 and 2.62 times respectively), which is in line with the faster development seen in this study. In Addition, the percentage mortality of nymphs in this study is lower than in the previous study (14) and mortality rate also had to be lowered in the modelling effort to make the model predictions match field observations (15). These differences are likely explained by the methods used in the two studies. The cages used in the current study allowed larger more robust TOH plants to be used than the tubes used in the previous study (14), which the authors of the previous study acknowledged were not ideal, especially for the larger nymphs. This is also consistent with the documented effects that the host used in the study can have on SLF nymphal development (17). In addition, the higher humidity and condensation present in the tubes could have trapped the nymphs and prevented them from feeding normally.

Exposure to extreme temperatures also had effects on the weights of newly-molted SLF nymphs. First and third instar (female) nymphs exposed to extreme temperatures as part of alternating regimes had lower weights compared to nymphs in the comparable average constant temperature (**Table 4**). The lower weights were probably due to energy being diverted from development to recovery from thermal stress or production costs of protectants against further thermal stress (31). For higher temperatures exposures, another possible explanation is that developmental rate would increase with increasing temperature, which can result in smaller body sizes (32). The exception was that second instar females that developed in the 20/5°C regime weighed more than those that developed in the 15°C constant temperature. One possible explanation is that the larger nymphs present in the 20/5°C were the only ones that were able to survive, since mortality was very high. The lack of weight differences between treatments in the fourth instars probably has a lot to do with the small sample size. Evaluating fourth instars in the laboratory is difficult because they have very high host demands which necessitate frequent plant changes and much reduced numbers per cage. Thus, results obtained for fourth instars should not be used in predicting what may happen in the field or used in models.

There was variation in the timing of hatch among egg masses both within and among populations when held at 15°C (**Table 5**). There are many possible reasons for this variation: historical factors like local adaptation, temperatures experienced before collection, and maternal effects, genetic variation, oviposition time, or individual plastic variation (33–35). For example, the exact temperatures the eggs were exposed to before collection and when each egg mass was laid are not known and that could have affected hatch timing. There is likely a consistent hatching stage but variation in hatch timing for SLF. Embryos may develop at different rates until they reach hatching competence after completing diapause but stay in a reduced metabolic state while they wait for another cue to hatch (which could differ between populations). If the cue to hatch never comes the embryos may die when energy reserves are exhausted as was seen in earlier work with SLF eggs (36). There is also evidence that the resumption of embryonic development is controlled by the expression of a heat shock protein and that a chill period is required to start that expression (37). The variation in hatch timing will buffer populations from mass mortality if they hatch too early or late in highly variable environments. In areas where the growing season is shorter there could be a major advantage to hatching as early as conditions become favourable, thus allowing sufficient time to reach the adult stage and oviposit before conditions become unfavourable again. If the first laid eggs also tend to hatch first and many females at a particular location are killed by cold before they can lay, selection for faster nymphal development and earlier hatch could occur. The earlier hatch in the NJ population compared to the PA ones would be advantageous since the average monthly highs are 2°C warmer and the monthly lows are 1°C warmer (based on data obtained from https://www.worldweatheronline.com) than at the NJ site, effectively resulting in a shorter growing season. The SLF populations have not been present at these sites that many years so selection may not have occurred yet. Another possible scenario that could explain hatch differences is that the preferred hosts at a site may decline in quality and nymphs may have to use alternate hosts, both of which could affect maternal provisioning of the eggs and timing of oviposition (can grow slower on less preferred hosts). Either egg provisioning or oviposition timing could in turn affect hatch timing. The SLF has been in the Philadelphia area longer than either the NJ or VA sites used in this study. Further work to determine exactly when eggs enter and exit diapause and how temperature effects that is needed to be able to determine the underlying reasons for the differences in hatch timing.

Variation in time in instar at the two constant temperatures also varied between populations and by instar. Insect populations exhibit local adaptation or plasticity in their developmental responses to temperature and this can vary between life stages (38). These differences can be the result of changes in the developmental thresholds and/or thermal requirements to complete development (38). The rough estimates (based on only 2 temperatures) of T_min_ for the first and second instars suggested that there may be 1-2°C variation between populations. When compared to previous estimates of T_min_ (13°C for firsts and 12°C for seconds) that variation could be up to 3°C (14). There is up to 4°C difference between the third instar T_min_ from this study and what was previously reported (14). There is also evidence of phenotypic plasticity across all instars since the slopes of the reaction norms (thermal response lines) of the populations are not equal (**Figures 4**–**6**) and there is evidence of a genotype by environment interaction since the lines cross for the second and third instars (**Figures 5**, **6**; **Table 6**). When lines cross it indicates that the phenotypic responses of the genotypes present in the populations differ based on the temperatures they are exposed to; the PA population grew the slowest at 15°C and the fastest at 25°C. The relative responses of the populations are consistent with the USDA plant hardiness zones they come from; first instars from the NJ population from the coldest zone grew the fastest at cooler temperatures than the other populations. This is also consistent with the temperature that the first instar nymphs would be exposed to at these sites in April when they hatch: NJ high 15°C and low 5°C, PA high 18°C and low 6°C (data from https://www.worldweatheronline.com). The patterns are inconsistent with the predicted decrease in T_min_ and increase in thermal requirements as latitude increases (38, 39). But there are two other factors that may play a role, elevation and heat islands of big cities. Just as ambient temperatures decrease by 7°C per 10° latitude, they also decrease by 6°C per km increase of altitude (40). Urban heat islands in the Northeast average 7-9°C warmer than surrounding rural areas (41). The PA populations were from lower elevations than the other two populations and from Philadelphia, PA area where there is an urban heat island. A broader survey of populations from across the SLF current range would be needed to assess the full variation in thermal responses. However, even the variability documented in this study is large enough to have impacts on predicted phenology and potential risk of establishment especially in areas colder areas than previously considered at risk.

Accurate phenology models based on SLF’s thermal responses are necessary for predicting when monitoring needs to occur, when the right stage is present for application of control methods, and for estimating the risk of establishment across the US. The new information from this study on variation present within and among populations in thermal requirements for hatch and development, as well as ability of nymphs to develop when exposed to alternating periods of temperatures above and below developmental thresholds and favourable temperatures should be integrated into the existing phenology models that rely on the older data (14) and used when new models are developed. Further work assessing more populations from a broader range of geographic locations and climates is still needed to better refine regional phenology predictions, but the present data will at least provide a starting point for the needed refinements to the models.

## Data availability statement

The raw data supporting the conclusions of this article will be made available by the authors, without undue reservation.

## Author contributions

MK and DK conceived the study. MK and GH got the funding for the study. MK and DK collected the data. MK analysed the data, prepared the figures and tables, and wrote the paper. All authors edited the manuscript. MK revised the paper based on reviewer input. All authors contributed to the article and approved the submitted version.
